# Non‐Linear Dose–Response Relationship for Metformin in Japanese Patients With Type 2 Diabetes: Analysis of Irregular Longitudinal Data by Interpretable Machine Learning Models

**DOI:** 10.1002/prp2.70055

**Published:** 2025-02-05

**Authors:** Hayato Akimoto, Takuya Nagashima, Kimino Minagawa, Takashi Hayakawa, Yasuo Takahashi, Satoshi Asai

**Affiliations:** ^1^ Division of Pharmacology, Department of Biomedical Sciences Nihon University School of Medicine; Oyaguchi‐Kamicho 30‐1 Itabashi‐ku Tokyo Japan; ^2^ Division of Genomic Epidemiology and Clinical Trials, Clinical Trials Research Center Nihon University School of Medicine Itabashi‐ku Tokyo Japan

**Keywords:** dose–response, interpretable machine learning, metformin, real‐world data

## Abstract

The dose–response relationship between metformin and change in hemoglobin A1c (HbA1c) shows a maximum at 1500–2000 mg/day in patients with type 2 diabetes (T2D) in the U.S. In Japan, there is little evidence on the HbA1c‐lowering effect of high‐dose metformin because the maintenance and maximum doses of metformin were raised in 2010. The aim of this study was to investigate whether there is saturation of the dose–response relationship for metformin in Japanese T2D patients. Longitudinal clinical information of T2D patients was extracted from electronic medical records. Supervised machine learning models with random effect were constructed to predict change in HbA1c: generalized linear mixed‐effects models (GLMM) with/without a feature selection and combining tree‐boosting with Gaussian process and mixed‐effects models (GPBoost). GPBoost was interpreted by SHapley Additive exPlanations (SHAP) and partial dependence. GPBoost had better predictive performance than GLMM with/without feature selection: root mean square error was 0.602 (95%CI 0.523–0.684), 0.698 (0.629–0.774) and 0.678 (0.609–0.753), respectively. Interpretation of GPBoost by SHAP and partial dependence suggested that the relationship between the daily dose of metformin and change in HbA1c is non‐linear rather than linear, and the HbA1c‐lowering effect of metformin reaches a maximum at 1500 mg/day. Interpretation of GPBoost, a non‐linear supervised machine‐learning algorithm, suggests that there is saturation of the dose–response relationship of metformin in Japanese patients with T2D. This finding may be useful for decision‐making in pharmacotherapy for T2D.

## Graphical abstract

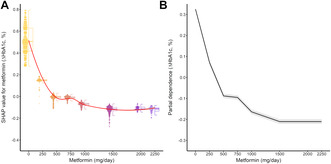

**Abbreviations**
ACE: angiotensin converting enzyme
ALT: alanine aminotransferase
ARB: angiotensin receptor blocker
AST: aspartate aminotransferase
BMI: body mass index
BUN: blood urea nitrogen
DPP‐4: dipeptidyl peptidase‐4
eGFR: estimated glomerular filtration rate
FPG: fasting plasma glucose
GLMM: generalized linear mixed‐effects model
GPBoost: combining tree‐boosting with Gaussian process and mixed‐effects model
HbA1C: hemoglobin A1c
NUSM's CDW: The Nihon University School of Medicine's Clinical Data Warehouse
SDA: serotonin dopamine antagonist
SGLT2: sodium‐glucose cotransporter 2
SHAP: SHapley Additive exPlanations
T2D: type 2 diabetes

## 1 Introduction

Metformin, one of the oldest oral hypoglycemic agents, is used as first‐line treatment for type 2 diabetes (T2D) worldwide. The glycated hemoglobin (HbA1c)‐lowering effect of metformin shows a dose‐dependent relationship from 500 up to 1500 mg/day, and reaches a maximum at 1500–2000 mg/day, as reported in several double‐blind, placebo‐controlled trials in the U.S. [1, 2] In Japan, the maintenance and maximum doses of metformin were increased to 750–1500 and 2250 mg/day, respectively, in May 2010 [3]. For this reason, few studies in Japan have evaluated the dose–response relationship between metformin and HbA1c, particularly at high doses (≥ 1000 mg/day). In T2D patients treated with metformin increased to 2250 mg/day, the daily profile of blood glucose levels tends to improve at 2250 mg/day compared to 1500 mg/day, but there is no significant difference between the two daily doses [4]. In contrast, Odawara et al. reported that HbA1c level was decreased in patients whose metformin daily dose was increased to 2250 mg/day for at least 12 weeks [5]. Although the dose–response relationship of metformin with glycemic control is inconsistent, if there is saturation of the dose–response relationship of metformin within the approved dose range (i.e., a non‐linear relationship), this might not only clarify the optimal daily dose of metformin, but also avoid the occurrence of dose‐dependent side effects such as a decrease in vitamin B12 [6].

Since the incidence of remission in Japanese T2D patients is 10.5 per 1000 person‐years and the remission rate is lower in patients with a longer duration of T2D [7], patients with T2D in real‐world settings continue receiving pharmacotherapy for many years. Because it has been reported that the HbA1c‐lowering effect of metformin wanes and HbA1c returns to baseline within 5 years [8], it is necessary to consider not only the relationship between the daily dose of metformin and HbA1c but also the duration of T2D when evaluating the HbA1c‐lowering effect of metformin. However, almost all previous studies that investigated the dose–response relationship between metformin and HbA1c had a follow‐up period of 1 year or less [1, 2, 5, 9], and, to our knowledge, there are no long‐term studies of the dose‐dependent HbA1c‐lowering effect of metformin in a real‐world setting. T2D patients with regular visits for diabetes care have better glycemic control, but the number of those with regular visits tends to be lower than that of those with irregular visits [10]. Thus, electronic medical records on T2D and its pharmacotherapy are irregularly collected at hospitals [11]. To evaluate the long‐term HbA1c‐lowering effect of metformin in real‐world settings, it is important to analyze longitudinal information on T2D patients accounting for irregular visits. Therefore, the aim of the present study was to investigate whether there is saturation of the dose–response relationship between metformin and change in HbA1c level (∆HbA1c) in Japanese T2D patients, using irregular longitudinal data.

## 2 Materials and Methods

### 2.1 Data Source

This study utilized electronic medical records from the Nihon University School of Medicine's Clinical Data Warehouse (NUSM's CDW) between April 1, 2004 and December 1, 2023. NUSM's CDW is a centralized data repository that integrates separate databases, including patient demographics, diagnoses, prescriptions and laboratory data, from the hospital information systems. To protect patient privacy, patient IDs are replaced by anonymous identifiers in all databases of the CDW, and the data in NUSM's CDW are mutually linked by the anonymized IDs. This study was approved by the Ethics Committee of NUSM (approval number: 2024‐05), and the study was conducted in compliance with the Ethical Guidelines for Medical and Health Research Involving Human Subjects of the Ministry of Education, Culture, Sports, Science and Technology and the Ministry of Health, Labour and Welfare, Japan.

### 2.2 Study Population

**FIGURE 1:**
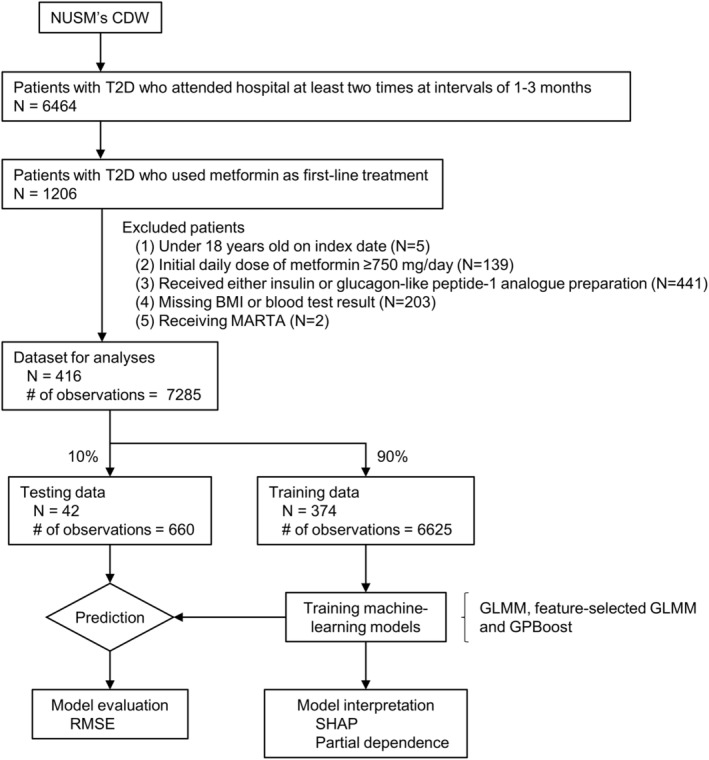
Sample size flow.

Sample size flow is shown in Figure 1. Data on Japanese patients with T2D (International classification of disease 10 codes are shown in Table S1) who had HbA1c ≥ 6.5% on two separate tests and had not been prescribed any hypoglycemic agent within the past 6 months were extracted from NUSM's CDW. The extracted patients were then condensed to patients who were started on metformin monotherapy at ≤ 500 mg/day, and the start date of metformin in these patients was regarded as the index date. Of these patients, patients who attended the hospital at intervals of at least 1 month (up to 3 months) were selected because HbA1c reflects the average blood glucose level over the past 1–3 months [12, 13], and the patients were followed up as long as they continued to attend during this interval. Patients who met the following exclusion criteria were excluded, and then the clinical information from the remaining T2D patients (*N* = 416) were obtained for analyses.

Excluded patients

1. Under 18 years old on index date
2. Started metformin at dose ≥ 750 mg/day
3. Received either insulin or a glucagon‐like peptide‐1 analogue preparation within all follow‐up periods, because the daily dose of these drugs is not recorded in NUSM's CDW
4. Missing body mass index (BMI) or any blood test results including hemoglobin, aspartate aminotransferase (AST), alanine aminotransferase (ALT), blood urea nitrogen (BUN) and estimated glomerular filtration rate (eGFR)
5. Received the following atypical antipsychotics within all follow‐up periods: clozapine, olanzapine, quetiapine and asenapine

### 2.3 Changes in HbA1c Level as Output and 50 Features

To assess the effect of the daily dose of metformin in the past 1–3 months on HbA1c level, ∆HbA1c between time points *t*_*i,j*_ and *t*_*i,j*−*1*_ in the *i*th patient were considered as an output, and the daily dose of metformin at *t*_*i,j*−*1*_ was considered as a feature (Figure S1A), where *i* = 1, 2, …, *N*, and *j* = 1 (i.e., index date), 2, …, *J*_*i*_, *N* is the number of patients, and *J*_*i*_ is the number of visits in the *i*th patient. In addition, age, sex (binary), BMI at first visit, estimated duration of T2D (from initial diagnosis to *t*_*i,j*−*1*_), number of oral hypoglycemic agents, six blood test results (previous HbA1c (HbA1c at *t*_*i,j*−*1*_), hemoglobin, AST, ALT, BUN and eGFR), daily dose of 21 oral hypoglycemic agents (three alpha‐glucosidase inhibitors, six dipeptidyl peptidase‐4 (DPP‐4) inhibitors, three glinides, five sodium‐glucose cotransporter 2 (SGLT2) inhibitors, three sulfonylureas, and pioglitazone; Table S2), presence/absence of six comorbidities (hypertension, dyslipidemia, hyperthyroidism, hypothyroidism, chronic kidney disease, and chronic liver disease; Table S1) and use/non‐use of 11 drug classes (angiotensin receptor blockers (ARBs), angiotensin converting enzyme (ACE) inhibitors, calcium blockers, selective beta‐1 blockers, alpha blockers, loop diuretics, thiazide diuretics, statins, fibrates, serotonin dopamine antagonists (SDAs), and steroids for systemic use; Table S3) at *t*_*i,j‐1*_ were also treated as features. As shown in Figure S1B, ∆HbA1c between *t*_*i,j*_ and *t*_*i,j‐1*_, and the 50 features at *t*_*i,j‐1*_ in the 416 patients were converted to data in long format, and the data were randomly split into training data for the development of machine‐learning models (90%; *N* = 374) and testing data for model evaluation (10%; *N* = 42).

### 2.4 Construction of Machine‐Learning Models and Model Evaluation

Three supervised machine‐learning models were constructed from the longitudinal data: (1) generalized linear mixed‐effects models with both a random intercept and random slope (GLMM), (2) feature‐selected GLMM using backward elimination method, and (3) combining tree‐boosting with Gaussian process and mixed‐effects models (GPBoost) for regression, which allows the fixed‐effects function to be modeled in a non‐linear manner and results in higher predictive accuracy compared to traditional linear mixed effect models [14]; that is, GPBoost can express the non‐linear dose–response relationship between metformin and ∆HbA1c. All the supervised machine‐learning models were developed using R software (version 4.1.2; R Foundation for Statistical Computing, Vienna, Austria).

Consider a sample of the 416 patients with T2D, let *Y*_*i,j*_ and *x*_*i,j*−*1*_ denote ∆HbA1c between *t*_*i,j*_ and *t*_*i,j*−*1*_ and the 50 features observed at *t*_*i,j*−*1*_ as fixed effects, respectively. In addition, patient ID was treated as a random effect. GLMM is shown in Equation (1):

(1) $$\begin{array}{c} Y_{i,j}=\begin{array}{c} \beta_{0}+x_{i,j-1}∙\beta +b_{0,i}+{\text{Duration}}_{i,j-1}∙b_{1,i}+\varepsilon_{i,j-1},\\ b_{k,i}\overset{\mathrm{i.i.d}}{\sim }N\left(0,\sigma_{k}^{2}\right),k\in \left\{0,1\right\},\varepsilon_{i,j-1}\overset{\mathrm{i.i.d}}{\sim }N\left(0,\sigma_{\varepsilon }^{2}\right) \end{array} \end{array}$$

where *β*_*0*_ is the intercept, *β* is the vector of coefficients of the fixed effects, *b*_*0,i*_ is the random intercept, *b*_*1,i*_ is the random slope for *Duration*_*i,j*−*1*_, *Duration*_*i,j*−*1*_ denotes the estimated duration of T2D from initial diagnosis to *t*_*i,j*−*1*_, and *ε*_*i,j*−*1*_ are residual terms for the *i*th patient with T2D. The GPBoost algorithm assumes that the response variable *Y*_*i,j*_ is the sum of a potentially non‐linear tree‐ensemble fixed effects function *F(∙)* and the random effects, as shown in Equation (2):

(2) $$\begin{array}{c} Y_{i,j}=\begin{array}{c} F\left(x_{i,j-1}\right)+b_{0,i}+{\text{Duration}}_{i,j-1}∙b_{1,i}+\varepsilon_{i,j-1},\\ b_{k,i}\overset{\mathrm{i.i.d}}{\sim }N\left(0,\sigma_{k}^{2}\right),k\in \left\{0,1\right\},\varepsilon_{i,j-1}\overset{\mathrm{i.i.d}}{\sim }N\left(0,\sigma_{\varepsilon }^{2}\right) \end{array} \end{array}$$

When constructing GPBoost using the R gpboost package, we ran subject‐wise 10‐fold cross‐validation to avoid identity confounding [15], and the following six hyperparameters were optimized by grid search: number of boosting iterations (“nrounds”), learning rate (“learning_rate”), number of leaves in a tree (“num_leaves”), maximal depth of a tree (“max_depth”), minimal number of samples per leaf (“min_data_in_leaf”), and L2 regularization (“lambda_l2”). The optimized hyperparameters are shown in Table S4. GLMM and feature‐selected GLMM were constructed using the training data and the R lme4 and lmerTest packages, because there are no hyperparameters to be tuned. In the backward elimination method, the significance level which is used to determine which fixed and random effects should be removed from a GLMM was set at 0.05, respectively. To evaluate the predictive performance of these machine‐learning models, the root mean square error (RMSE) for the training and testing data was calculated, respectively. The 95% confidence intervals (95%CIs) of RMSE were estimated with a percentile bootstrap method [16].

### 2.5 Non‐linear Dose–Response Relationship Between Metformin and Change in HbA1c Level

To depict a non‐linear dose–response relationship between daily dose of metformin and ∆HbA1c, SHapley Additive exPlanations (SHAP) value and partial dependence were used. The former is an algorithm for interpreting the output of machine‐learning models, and was calculated using the R SHAPforxgboost package. The latter is a technique to understand the relationship between a feature (or a set of features) of interest and the predicted output of a machine‐learning model, and was calculated as the marginal effect of the feature on the predicted output while averaging out the effects of all other features. The one‐way partial dependence plot can show whether the relationship between a feature of interest and the predicted output is linear or more complex. In addition, in order to detect factors modifying the lowering effect of metformin on HbA1c, two‐way partial dependence plots were depicted, and Friedman's H statistic was calculated to estimate two‐way interaction strength using the R flashlight package. The H statistic tends to be between 0 and 1; this statistic is 0 if there is no interaction and 1 if two features of interest only affect the prediction through an interaction.

## 3 Results

### 3.1 Patients' Characteristics

Longitudinal data with irregular observation times of the 416 eligible patients with T2D (total number of observations = 7285) were obtained from NUSM's CDW. Their clinical characteristics are presented in Table 1. Age of T2D patients at the index date was 57.8 ± 12.5 years (mean ± SD), and many T2D patients were in their 50s and 60s regardless of sex (Figure S2). BMI of T2D patients was 26.0 ± 5.1 kg/m^2^. Median HbA1c level at the index date was 8.2% (interquartile range (IQR) 7.4%–9.6%). Of the 416 patients, 46 patients were started on metformin at 250 mg/day and the remaining patients at 500 mg/day. After starting metformin, HbA1c level was maintained at approximately 7.0% throughout the follow‐up period, while the daily dose of metformin was increased and other oral hypoglycemic agents were added. Additionally, the prevalence of the six comorbidities and 11 drug classes tended to increase with estimated duration of T2D, particularly for hypertension, dyslipidemia and drug classes for these comorbidities such as ARBs, calcium blockers and statins.

**TABLE 1:** Long‐term trends in clinical characteristics of Japanese patients with type 2 diabetes.

Clinical characteristics	Index date (*N* = 416)	1 year (*N* = 224)	2 years (*N* = 170)	4 years (*N* = 108)	6 years (*N* = 68)	8 years (*N* = 38)	10 years (N = 22)
Age (years), mean (SD)	57.8 (12.5)	57.5 (11.6)	58.9 (11.4)	61.2 (11.6)	63.9 (10.4)	67.5 (10.2)	66.6 (10.1)
Male, *N* (%)	266 (63.9)	138 (61.6)	105 (61.8)	65 (60.2)	45 (66.2)	19 (50.0)	11 (50.0)
BMI (kg/m^2^), mean (SD)	26.0 (5.1)	26.2 (5.3)	25.9 (5.1)	26.0 (4.8)	25.9 (4.5)	25.6 (4.2)	26.2 (4.9)
Estimated duration of T2D (months), median (IQR)	0.0 (0.0–4.4)	14.6 (13.5–18.2)	27.0 (25.4–30.3)	50.7 (49.7–54.5)	75.5 (74.5–78.9)	100.7 (98.9–103.5)	127.7 (123.6–128.3)
Blood test result, median (IQR)							
HbA1c (%)	8.2 (7.4–9.6)	7.0 (6.6–7.7)	7.1 (6.6–7.7)	7.1 (6.8–7.8)	7.0 (6.6–7.7)	7.0 (6.7–7.5)	7.0 (6.8–7.7)
Hemoglobin (g/dL)	14.5 (13.4–15.5)	14.2 (13.2–15.4)	14.2 (13.1–15.2)	14.0 (12.8–15.1)	13.9 (12.9–15.4)	13.4 (12.5–14.6)	13.1 (12.2–14.7)
AST (IU/L)	23.0 (18.0–34.2)	22.0 (18.0–29.2)	21.5 (18.0–28.0)	22.0 (19.0–30.0)	20.5 (17.0–30.0)	21.0 (18.2–27.0)	20.5 (15.2–23.0)
ALT (IU/L)	27.0 (18.0–46.6)	23.5 (16.0–39.0)	24.0 (16.0–40.0)	23.0 (17.0–39.0)	20.0 (15.8–34.2)	19.0 (14.0–28.0)	17.5 (12.2–25.2)
BUN (mg/dL)	14.3 (11.6–16.8)	13.6 (11.4–16.4)	14.2 (11.4–17.0)	14.8 (12.4–17.3)	15.4 (12.0–17.7)	14.9 (12.8–17.0)	14.6 (12.1–18.9)
eGFR (mL/min/1.73m^2^)	78.5 (65.7–93.7)	75.7 (64.9–93.2)	76.1 (64.6–88.4)	75.3 (63.9–88.1)	71.3 (63.3–82.2)	69.4 (59.7–80.5)	71.4 (58.2–83.9)
Number of OHAs, median (IQR)	1 (1–1)	2 (1–2)	2 (1–3)	2 (2–4)	3 (2–4)	4 (2–5)	4 (3–5)
Metformin [mg/day], *N*							
0	0	4	4	0	0	0	0
250	46	2	1	0	1	0	0
500	370	98	59	29	17	11	5
750	0	54	45	25	11	8	7
1000	0	45	38	33	27	11	6
1500	0	19	20	17	10	6	4
2000	0	0	1	4	1	1	0
2250	0	2	2	0	1	1	0
Other OHAs, *N*							
[Daily dose, mean (SD)]							
Acarbose	0	3	3	3	2	2	1
[mg/day]	— (—)	300.0 (0.0)	300.0 (0.0)	300.0 (0.0)	300.0 (0.0)	300.0 (0.0)	300.0 (—)
Voglibose	0	11	11	6	7	4	3
[mg/day]	— (—)	0.7 (0.2)	0.7 (0.2)	0.8 (0.2)	0.8 (0.2)	0.7 (0.2)	0.7 (0.2)
Miglitol	0	10	10	9	6	6	4
[mg/day]	— (—)	150.0 (0.0)	157.5 (23.7)	150.0 (0.0)	150.0 (0.0)	133.3 (40.8)	106.2 (51.5)
Alogliptin	0	6	5	9	7	8	4
[mg/day]	— (—)	20.8 (6.5)	20.0 (6.8)	19.4 (6.6)	19.6 (6.7)	21.9 (5.8)	25.0 (0.0)
Saxagliptin	0	0	0	3	1	0	0
[mg/day]	— (—)	— (—)	— (—)	5.0 (0.0)	5.0 (—)	— (—)	— (—)
Sitagliptin	0	32	39	28	20	16	13
[mg/day]	— (—)	60.9 (21.0)	55.8 (17.6)	58.0 (18.1)	55.0 (15.4)	53.1 (12.5)	57.7 (18.8)
Teneligliptin	0	13	13	7	6	4	2
[mg/day]	— (—)	24.6 (8.8)	24.6 (8.8)	20.0 (0.0)	20.0 (0.0)	25.0 (10.0)	20.0 (0.0)
Vildagliptin	0	29	23	23	21	14	8
[mg/day]	— (—)	96.6 (12.9)	95.7 (14.4)	97.8 (10.4)	100.0 (0.0)	100.0 (0.0)	100.0 (0.0)
Linagliptin	0	7	6	3	3	3	1
[mg/day]	— (—)	5.0 (0.0)	5.0 (0.0)	5.0 (0.0)	5.0 (0.0)	5.0 (0.0)	5.0 (—)
Nateglinide	0	2	3	3	3	3	1
[mg/day]	— (—)	270.0 (0.0)	270.0 (0.0)	270.0 (0.0)	210.0 (103.9)	210.0 (103.9)	270.0 (—)
Mitiglinide	0	3	2	1	0	0	0
[mg/day]	— (—)	30.0 (0.0)	30.0 (0.0)	30.0 (—)	— (—)	— (—)	— (—)
Repaglinide	0	3	2	2	1	1	1
[mg/day]	— (—)	1.0 (0.4)	0.8 (0.0)	0.5 (0.4)	0.2 (—)	0.2 (—)	0.2 (—)
Ipragliflozin	0	2	3	4	3	1	1
[mg/day]	— (—)	50.0 (0.0)	50.0 (0.0)	50.0 (0.0)	50.0 (0.0)	50.0 (—)	50.0 (—)
Empagliflozin	0	3	1	0	2	0	1
[mg/day]	— (—)	10.0 (0.0)	10.0 (—)	— (—)	15.0 (7.1)	— (—)	10.0 (—)
Canagliflozin	0	6	5	5	4	4	2
[mg/day]	— (—)	100.0 (0.0)	100.0 (0.0)	100.0 (0.0)	100.0 (0.0)	100.0 (0.0)	100.0 (0.0)
Dapagliflozin	0	6	6	7	2	0	0
[mg/day]	— (—)	5.8 (2.0)	5.8 (2.0)	6.4 (2.4)	5.0 (0.0)	— (—)	— (—)
Luseogliflozin	0	2	2	1	0	0	0
[mg/day]	— (—)	2.5 (0.0)	2.5 (0.0)	2.5 (—)	— (—)	— (—)	— (—)
Glimepiride	0	33	31	24	19	14	9
[mg/day]	— (—)	1.6 (1.3)	1.4 (1.1)	1.3 (0.8)	1.4 (1.1)	1.1 (0.6)	1.0 (0.5)
Gliclazide	0	19	23	22	18	15	12
[mg/day]	— (—)	50.0 (27.7)	45.2 (29.5)	44.1 (27.4)	47.2 (29.1)	40.7 (30.3)	44.2 (32.9)
Glibenclamide	0	12	10	8	4	3	3
[mg/day]	— (—)	4.1 (2.6)	4.1 (2.8)	3.1 (3.1)	3.8 (3.1)	1.2 (0.0)	1.2 (0.0)
Pioglitazone	0	18	17	12	8	5	3
[mg/day]	— (—)	16.2 (6.9)	16.3 (7.1)	18.8 (8.8)	20.6 (7.8)	21.0 (8.2)	20.0 (8.7)
Comorbidity							
Hypertension	76 (18.3)	131 (58.5)	102 (60.0)	75 (69.4)	46 (67.6)	30 (78.9)	18 (81.8)
Dyslipidemia	62 (14.9)	105 (46.9)	87 (51.2)	61 (56.5)	44 (64.7)	29 (76.3)	17 (77.3)
Hyperthyroidism	2 (0.5)	5 (2.2)	5 (2.9)	4 (3.7)	3 (4.4)	3 (7.9)	1 (4.5)
Hypothyroidism	2 (0.5)	10 (4.5)	8 (4.7)	5 (4.6)	3 (4.4)	2 (5.3)	2 (9.1)
Chronic liver disease	3 (0.7)	8 (3.6)	7 (4.1)	3 (2.8)	3 (4.4)	1 (2.6)	1 (4.5)
Chronic kidney disease	3 (0.7)	12 (5.4)	9 (5.3)	7 (6.5)	4 (5.9)	2 (5.3)	2 (9.1)
Other drug classes							
ARBs	51 (12.3)	80 (35.7)	60 (35.3)	49 (45.4)	29 (42.6)	20 (52.6)	12 (54.5)
ACE inhibitors	1 (0.2)	9 (4.0)	9 (5.3)	6 (5.6)	4 (5.9)	3 (7.9)	2 (9.1)
Calcium channel blockers	43 (10.3)	73 (32.6)	57 (33.5)	46 (42.6)	32 (47.1)	20 (52.6)	12 (54.5)
Selective β_1_ blockers	11 (2.6)	11 (4.9)	11 (6.5)	9 (8.3)	7 (10.3)	3 (7.9)	2 (9.1)
α blockers	6 (1.4)	5 (2.2)	4 (2.4)	4 (3.7)	0 (0.0)	0 (0.0)	1 (4.5)
Loop diuretics	6 (1.4)	13 (5.8)	11 (6.5)	8 (7.4)	5 (7.4)	4 (10.5)	4 (18.2)
Thiazide diuretics	4 (1.0)	8 (3.6)	8 (4.7)	4 (3.7)	2 (2.9)	1 (2.6)	0 (0.0)
Statins	46 (11.1)	86 (38.4)	73 (42.9)	56 (51.9)	40 (58.8)	27 (71.1)	16 (72.7)
Fibrates	6 (1.4)	11 (4.9)	9 (5.3)	6 (5.6)	9 (13.2)	5 (13.2)	1 (4.5)
SDAs	5 (1.2)	5 (2.2)	4 (2.4)	2 (1.9)	0 (0.0)	0 (0.0)	0 (0.0)
Steroids (systemic use)	32 (7.7)	25 (11.2)	18 (10.6)	11 (10.2)	7 (10.3)	6 (15.8)	4 (18.2)

Abbreviations: ACE, angiotensin converting enzyme; ALT, alanine aminotransferase; ARB, angiotensin receptor blocker; AST, aspartate aminotransferase; BUN, blood urea nitrogen; eGFR, estimated glomerular filtration rate; HbA1c, hemoglobin A1c; IQR, interquartile range; IU, international unit; OHA, oral hypoglycemic agent; SD, standard deviation; SDA, serotonin dopamine antagonist.

### 3.2 Evaluation of Three Machine‐Learning Models for Prediction of Changes in HbA1c Level

**FIGURE 2:**
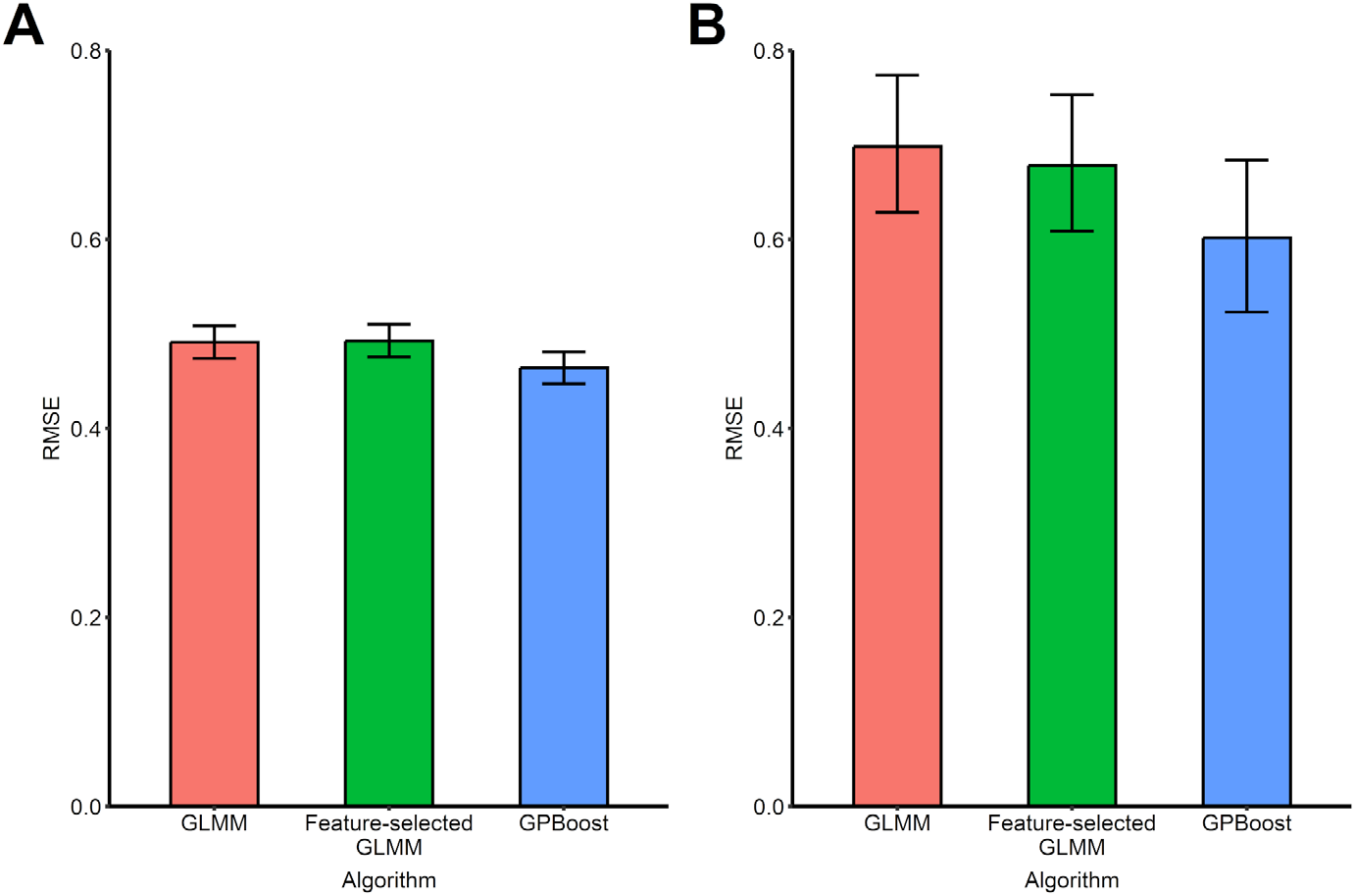
Comparison of predictive performance among three machine‐learning models on training and testing data. Error bar indicates 95% CI. 95% CI was defined as the 50th to 1950th quantiles of the distribution of the 2000 bootstrap samples.

The RMSE values of GLMM, feature‐selected GLMM and GPBoost are shown in Figure 2. In the training data, GPBoost (0.464; 95%CI 0.448–0.481) had the best predictive performance for ∆HbA1c compared to GLMM (0.491; 0.474–0.508) and feature‐selected GLMM (0.493; 0.476–0.510). Similarly, GPBoost (0.602; 0.523–0.684) had the best predictive performance also in the testing data: GLMM (0.698; 0.629–0.774) and feature‐selected GLMM (0.678; 0.609–0.753).

### 3.3 Relationship Between Daily Dose of Metformin and Lowering Effect on HbA1c

GLMM was constructed using all 50 fixed effects, the random intercept and slope for estimated duration of T2D, whereas 19 fixed effects, the random intercept and slope were selected by backward elimination method in feature‐selected GLMM. As shown in Table 2, the daily dose of metformin was significantly associated with the lowering effect of HbA1c in the two GLMMs irrespective of the presence/absence of feature selection. Associations of all the fixed effects with ∆HbA1c are shown in Table S5.

**TABLE 2:** Association of daily dose of metformin with change in HbA1c level in two linear algorithms.

Algorithm	Coefficient^a^	Standard error	df	*t*	*p*
GLMM	−0.234	0.0145	3994	−16.18	< 0.001
Feature‐selected GLMM	−0.231	0.0142	3894	−16.31	< 0.001

Abbreviations: df, degrees of freedom; GLMM, generalized linear mixed effects model.

^a^ Regression coefficients are expressed as change in HbA1c level per metformin 500 mg/day adjusted for age, sex, BMI, estimated duration of T2D, blood test results, number of oral hypoglycemic agents, daily dose of oral hypoglycemic agents, presence/absence of comorbidities and use/non‐use of other drug classes.

**FIGURE 3:**
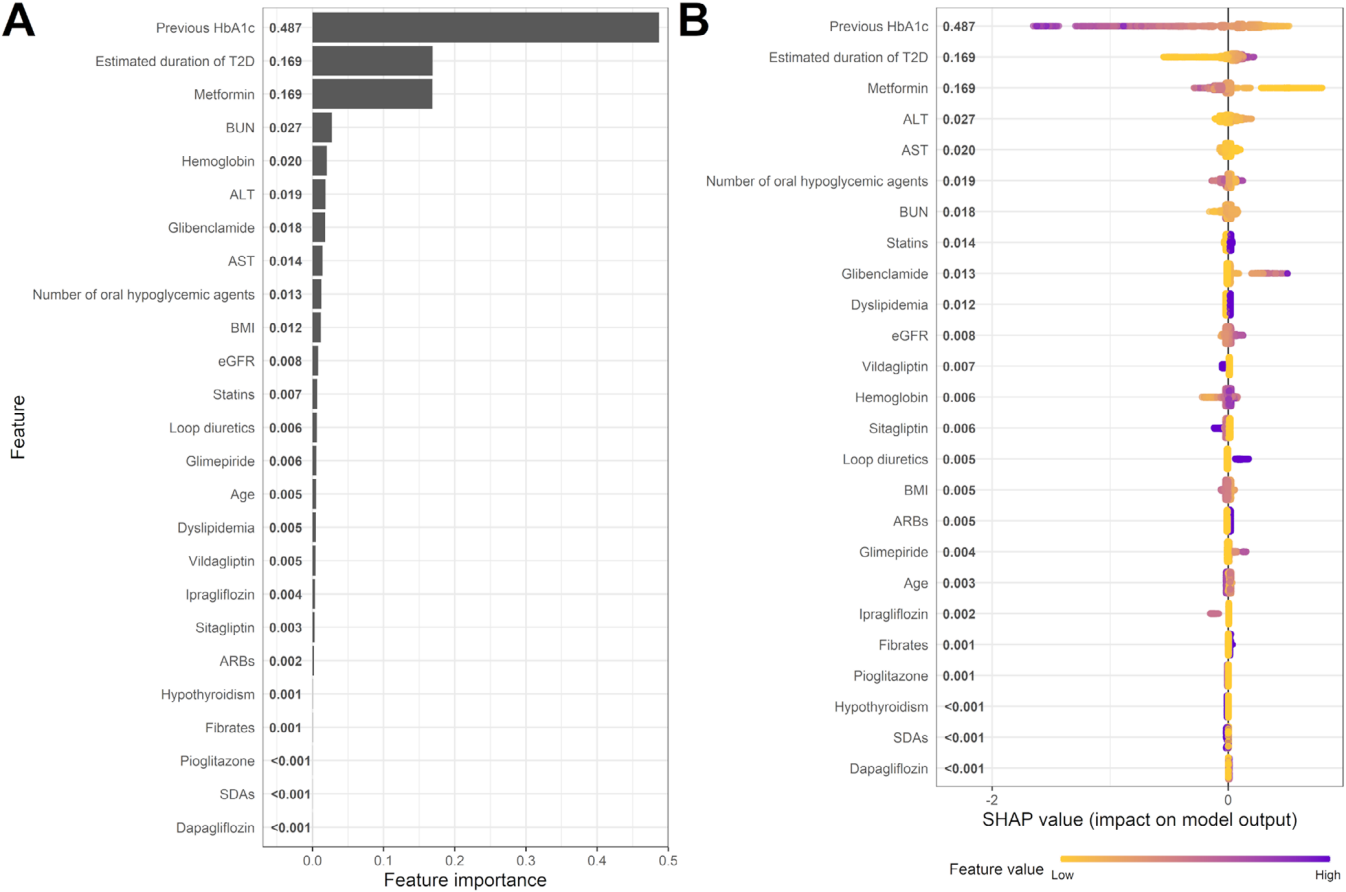
Important predictors of ΔHbA1c in GPBoost algorithm. Panels A and B are feature importance and SHAP summary plots, respectively. Only non‐zero features are shown in both panels A and B.

To identify the features that contribute to prediction of ∆HbA1c in GPBoost, feature importance and SHAP summary plots are shown in Figure 3. The top three important features for prediction of ∆HbA1c were previous HbA1c, estimated duration of T2D and daily dose of metformin, followed by BUN, hemoglobin, ALT etc. (Figure 3A). The first three features appeared to play an important role in prediction of ∆HbA1c also in the SHAP summary plot. In Figure 3B, features are sorted from top to bottom by the mean value of absolute SHAP, which is shown to the right of each feature, and each dot represents an observation in a patient with T2D. For example, purple dots—which represent observations of high feature value—of the most important feature “previous HbA1c” had a negative impact on the model output, as the corresponding horizontal axis position had negative SHAP values. Previous HbA1c was the most important feature predicting the output, with higher HbA1c level at *t*_*i,j*−*1*_ indicating a greater change in ∆HbA1c between *t*_*i,j*−*1*_ and *t*_*i,j*_ in a negative direction. In addition, higher daily dose of metformin was also an important feature that changed ∆HbA1c in a negative direction.

**FIGURE 4:**
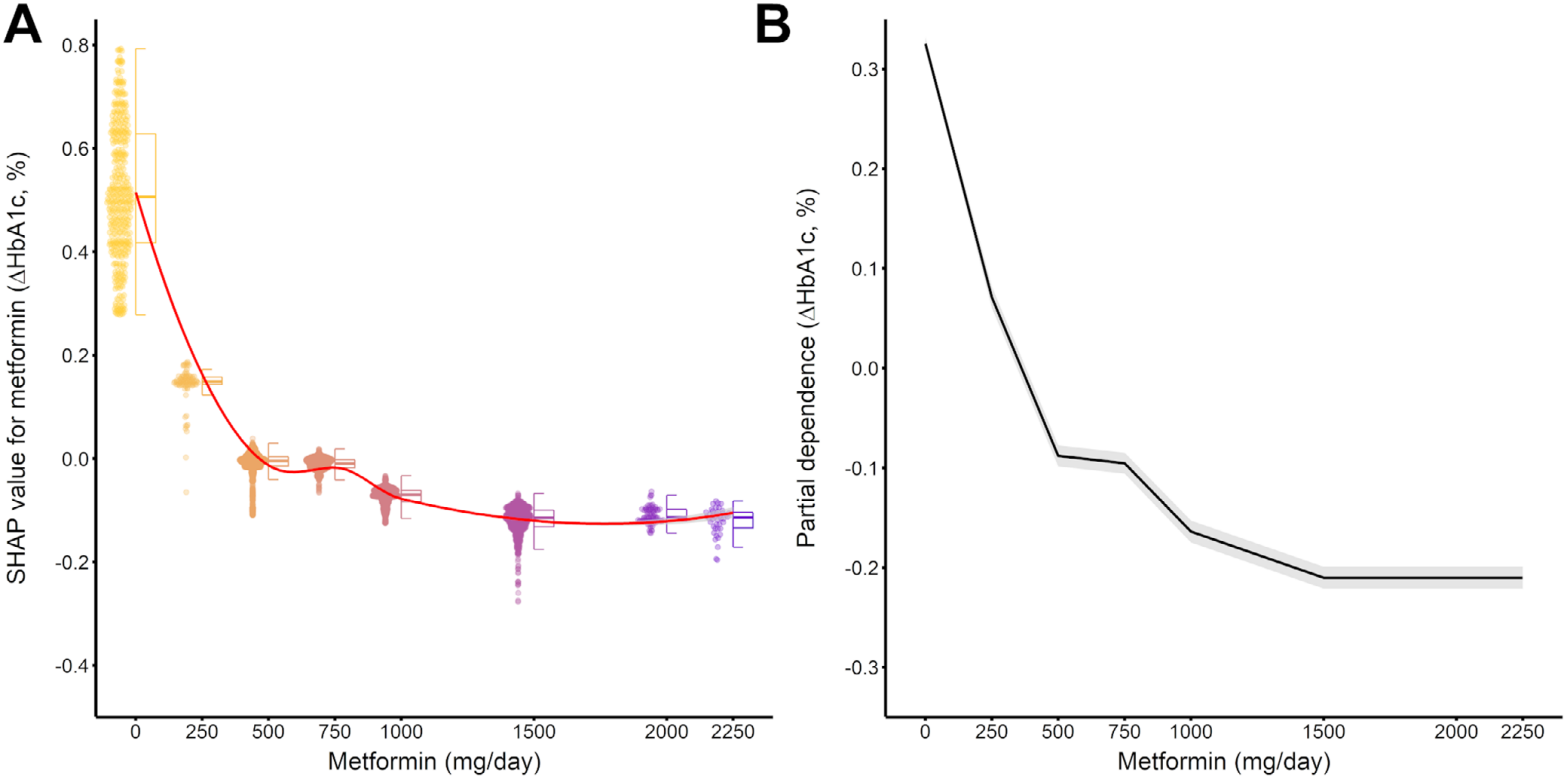
Non‐linear dose–response relationship between daily dose of metformin and ΔHbA1c. Panel A is the SHAP dependence plot (hybrid boxplot). Red line and gray ribbon indicate locally weighted scatterplot smoothing regression curve and the corresponding 95% CI, respectively. Panel B is the one‐way partial dependence plot to understand the effect of daily dose of metformin on ΔHbA1c. Black line and gray ribbon indicate partial dependence and the corresponding 95% CI, respectively.

The non‐linear dose–response relationship between metformin and ∆HbA1c is shown in Figure 4. Figure 4A is a hybrid boxplot plot of SHAP values for the daily dose of metformin in each observation. There was a dose‐dependent decrease in SHAP value with metformin in the range of 0–1500 mg/day, while SHAP value at 1500 mg/day was equivalent to those at 2000 and 2250 mg/day. Figure 4B is the one‐way partial dependence plot depicting the relationship between daily dose of metformin and ∆HbA1c. Partial dependence decreased as the daily dose of metformin increased in the range of 0–1500 mg/day, but partial dependence was equivalent above 1500 mg/day. As the maintenance and maximum doses of metformin were increased in May 2010, the data were split into before and after May 2010 based on the index date. SHAP dependence and partial dependence plots in gpboost, which were constructed for each data set, are shown in Figure S3. Also in gpboost constructed using only data after May 2010, SHAP and partial dependence showed dose‐dependent decreases in the range of 0–1500 mg/day.

### 3.4 Features Modifying the Lowering Effect of Metformin on HbA1c

**FIGURE 5:**
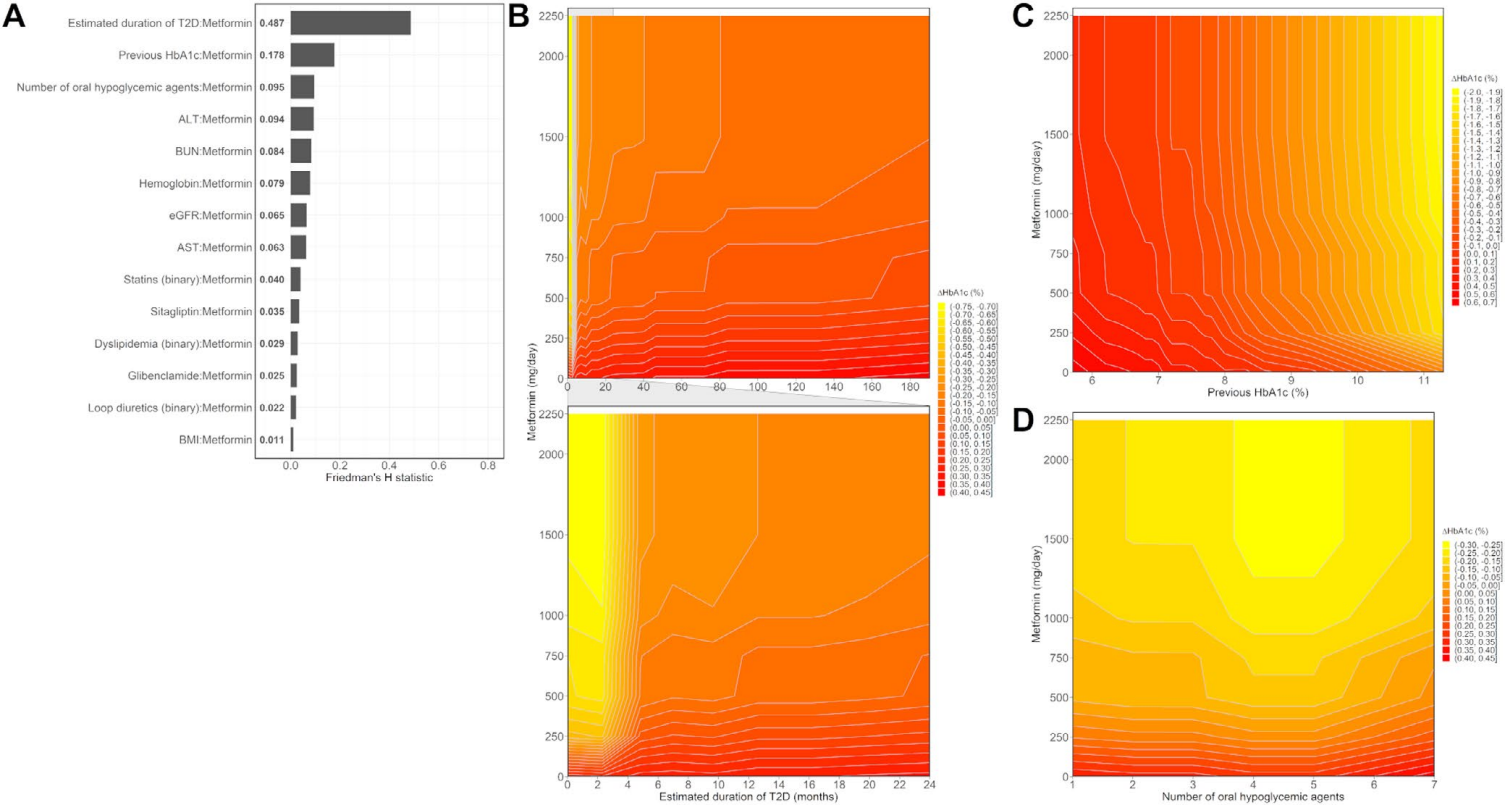
Interactions between daily dose of metformin and other features on ΔHbA1c. Friedman's H statistic between daily dose of metformin and the other features are shown in panel A. Two‐way partial dependence plots (filled contour plots) between daily dose of metformin and estimated duration of T2D, previous HbA1c, and number of oral hypoglycemic agents are shown in Panels B–D, respectively.

Interactions between metformin and other features on ∆HbA1c are shown in Figure 5A. Friedman's H statistic between metformin and each of the 14 features was non‐zero. Especially, the estimated duration of T2D most strongly modified the HbA1c‐lowering effect of metformin. The two‐way partial dependence plot (filled contour plot) between the daily dose of metformin and estimated duration of T2D is shown at the top of Figure 5B, and a plot zoomed in on estimated duration in the range from 0 to 24 months is shown at the bottom of Figure 5B. The yellow‐to‐red gradient in the two‐way partial dependence plot represents change in ∆HbA1c due to interactions between two features of interest. For example, partial dependence increased rapidly when metformin at a dose of 500 mg/day was maintained for 6 months from estimated duration of T2D of 0 month; that is, the longer the estimated duration of T2D, the more attenuated the HbA1c‐lowering effect of metformin. The two‐way partial dependence plot between daily dose of metformin and previous HbA1c is shown in Figure 5C. The higher the previous HbA1c, the greater the HbA1c‐lowering effect of metformin. ΔHbA1c slightly increased in a negative direction when two or more (up to five) oral hypoglycemic agents were combined, compared to metformin single use (Figure 5D). Estimated duration of T2D was the greatest modifier of the HbA1c‐lowering effect of metformin, followed by previous HbA1c level and number of oral hypoglycemic agents. Partial dependence plots of all 14 features that could modify the effect of metformin are shown in Figures S4 and S5.

## 4 Discussion

In the present study, three machine‐learning models were constructed for the prediction of ∆HbA1c in Japanese T2D patients who received metformin as first‐line treatment, to investigate whether there is saturation of the HbA1c‐lowering effect of metformin. GPBoost that was not assumed to be linear between the output and the features had the best predictive performance among these models. By interpreting GPBoost using SHAP and partial dependence, estimated duration of T2D, previous HbA1c level and daily dose of metformin were identified as the top three important predictors of ∆HbA1c during pharmacological treatment for T2D, and the first two features also modified the effect of metformin on HbA1c level. Several randomized controlled trials have reported that the HbA1c‐lowering effect of metformin varies with estimated duration of metformin use. The UK Prospective Diabetes Study Group has reported that HbA1c and fasting plasma glucose (FPG) levels decreased immediately after starting metformin in overweight patients with T2D followed up to 10 years, but both variables gradually increased thereafter, returning to baseline levels after 4–5 years [17]. Kahn et al. reported that HbA1c and FPG levels in more than 1000 patients with T2D at the start of metformin treatment decreased immediately after the initiation and returned to baseline levels after approximately 5 years [8]. In the present study, the interaction between daily dose of metformin and estimated duration of T2D, but not the duration of metformin use, was assessed as shown in Figure 5B, and depending on the daily dose of metformin, the effect of metformin at 500 mg/day on ΔHbA1c was close to zero when the estimated duration of T2D reached 40–50 months. Estimated duration of T2D roughly coincides with duration of metformin use, because 75% of T2D patients started metformin within 4.4 months after the initial diagnosis of T2D in the present study (Table 1). Previous HbA1c was the most important predictor of change in HbA1c level and was a modifier of the HbA1c‐lowering effect of metformin. In this study, irregular longitudinal data were generated to flexibly incorporate events (e.g., increase/decrease in daily dose of metformin, addition/deletion of other oral hypoglycemic agents, variations in blood test results, development of comorbidities and their treatment) that occasionally occur during pharmacotherapy for T2D into the ΔHbA1c prediction model. Therefore, the sequentially updated baseline HbA1c levels were treated as previous HbA1c levels. It is known that the baseline HbA1c level of patients who have been recruited into clinical trials strongly influences the HbA1C reduction following pharmacologic intervention [18]. Wilding et al. reported that baseline HbA1c level correlated with the changes in HbA1c level at 6 and 18 months in patients with T2D who had received metformin monotherapy as first‐line treatment and were started on second‐line treatment [19]. In Japan, baseline HbA1c was significantly associated with change in HbA1c level at 24 weeks in patients with T2D who received metformin [20], and the results of the present study supported the findings from these clinical studies. In summary, GPBoost included the findings of various clinical studies reported to date as important predictors, and is a realistic model for predicting change in HbA1c level in clinical practice.

GPBoost suggested that the dose–response relationship between the daily dose of metformin and change in HbA1c level is non‐linear; in other words, there is saturation of the dose–response relationship within the approved dose range (Figure 4). Several clinical studies have reported the maximal efficacy of metformin on HbA1c. Garber et al. observed that the HbA1c‐lowering effect of immediate‐release metformin reached a maximum at 2000 mg/day during 11 weeks of treatment, while the study was not designed to evaluate the clinical practice of titrating the dosage according to individual glycemic control [1]. Fujioka et al. reported that the HbA1c‐lowering effect of metformin reached a maximum at 1500 mg/day in patients with T2D who received an extended‐release formulation of metformin with increasing daily dose at weekly intervals until the target daily dose [2]. Furthermore, the findings of this study are consistent with the results of a dose–response study of metformin that are included in the prescribing information on GLUCOPHAGE and FORTAMET published by the FDA. Therefore, similar to the results of these clinical studies, the HbA1c‐lowering effect of metformin in Japanese patients with type 2 diabetes may become saturated within the approved dose range. Pharmacokinetic parameters of metformin vary in a dose‐dependent manner. Timmins et al. reported that in healthy volunteers, area under the plasma concentration‐time curve and maximum plasma concentration value of metformin at steady state dose‐dependently increased in the range of 500–2000 mg/day [21, 22]. Therefore, pharmacokinetics may not be responsible for the saturation of the dose–response relationship of metformin, with a maximum effect at 1500 mg/day. The antihyperglycemic effects of metformin have been thought to mainly arise from the control of hepatic gluconeogenesis, through both AMP‐activated protein kinase (AMPK)‐dependent and AMPK‐independent mechanisms in the liver. However, increasing evidence suggests a role of extrahepatic mechanisms [23]. Metformin exhibits gastrointestinal effects to lower the blood glucose via slowing of gastric emptying, secretion of GLP‐1 from L cells located in the small intestine, inhibition of luminal glucose and bile acid absorption, and alteration of the gut microbiota [24, 25, 26, 27]. Additionally, in the gut, metformin influx into the enterocyte via saturable apical transport and insufficient release through the basolateral membrane result in accumulation of metformin in the enterocyte. The high accumulation of metformin in the gut suggests that the gut acts as a reservoir of metformin [23]. In other words, as metformin lowers blood glucose level by hepatic and gastrointestinal mechanisms, metformin concentration in the gut as well as in the plasma may contribute to determine the its glucose‐lowering effect. However, as the concentrations of metformin in the plasma and gut are not recorded in NUSM's CDW, the association between the concentrations of metformin and HbA1c‐lowering effects cannot be assessed in this study. The complexity of the therapeutic action of metformin thus makes it difficult to explain the saturation of the dose–response relationship in terms of the mechanism of action of metformin. Therefore, factors that can adequately explain the saturation of the dose–response relationship of metformin remain unclear.

The present study has several limitations. First, there is a possibility of sampling bias because this study had a retrospective observational study design using non‐randomized data. Second, this study controlled for potential confounding factors that were available and measurable, but failed to adjust for non‐observed factors. For example, it is reported that improved medication adherence to prescribed oral hypoglycemic agents is associated with lower HbA1c levels [28]. However, it is not possible to adjust for this effect because NUSM's CDW does not record measures of medication adherence. Third, the medical information on the T2D patients in this study was collected from a single institution and the sample size was relatively small. Therefore, the machine learning models do not consider differences in technical interventions by healthcare providers and facilities as random effects, which may lead to bias in the results of the analysis. Furthermore, not all the T2D patients experienced high doses of metformin, with relatively few observations of 2000 and 2250 mg/day. This may compromise the accuracy of the machine‐learning models at high doses of metformin. However, the characteristics of the 416 Japanese T2D patients in this study, including age, BMI and sex ratio, are well matched to those of T2D patients in the National Health and Nutrition Survey [29] and a multicenter clinical study [5] conducted in Japan. Furthermore, the saturation of the metformin dose–response relationship revealed by interpreting machine learning models constructed from their electronic medical records supports the results of double‐blind, placebo‐controlled studies conducted in the U.S. [1] Therefore, we believe that the findings of this study have external validity. Finally, the database cannot access clinical information stored at other medical institutions. In this study, longitudinal clinical information on Japanese patients with T2D was extracted from NUSM's CDW. If some of these patients had continued to receive treatment at other medical institutions after attending our hospital several times, their subsequent clinical information could not be included in the analyses. However, the saturation of the metformin dose–response relationship revealed by interpreting GPBoost, which had the best predictive performance and is a realistic model for predicting changes in HbA1c level in clinical practice, may contribute to decision‐making in pharmacotherapy for T2D.

## Author Contributions

H.A., T.H. and S.A. designed the research, H.A., T.N. and K.M. acquired and analyzed the data, H.A. and Y.T. interpreted the analyzed results, and H.A. wrote the manuscript. All authors have read and agreed to its content and have approved the final manuscript for submission.

## Ethics Statement

The project was approved by the Ethics Committee of NUSM (approval number: 2024‐05).

## Consent

According to the Ministry of Health, Labor and Welfare of Japan, informed consent is not required for the present study which accessed anonymized and pooled patient information.

## Conflicts of Interest

The authors declare no conflicts of interest.

## Supporting information


Figure S1.

Table S1.

## Data Availability

The data that support the findings of this study are available on request from the corresponding author. The data are not publicly available due to privacy or ethical restrictions.
