# Supplementary material for: Non‐Linear Dose–Response Relationship for Metformin in Japanese Patients With Type 2 Diabetes: Analysis of Irregular Longitudinal Data by Interpretable Machine Learning Models
Source: Pharmacol Res Perspect. 2025 Feb 5;13(1):e70055. doi: 10.1002/prp2.70055 (PMC11797302; doi:10.1002/prp2.70055)
Supplement: Supplementary file 1 — Figure S1. [file PRP2-13-e70055-s001.docx]

***Supplementary Figures***

**Non-linear dose-response relationship for metformin in Japanese patients with type 2 diabetes: Analysis of irregular longitudinal data by interpretable machine learning models**

**Hayato Akimoto^1,2^, Takuya Nagashima^1,2^, Kimino Minagawa^2^, Takashi Hayakawa^1,2^, Yasuo Takahashi^2^ and Satoshi Asai^1,2^**

* Correspondence: Hayato Akimoto: [akimoto.hayato@nihon-u.ac.jp](mailto:akimoto.hayato@nihon-u.ac.jp)


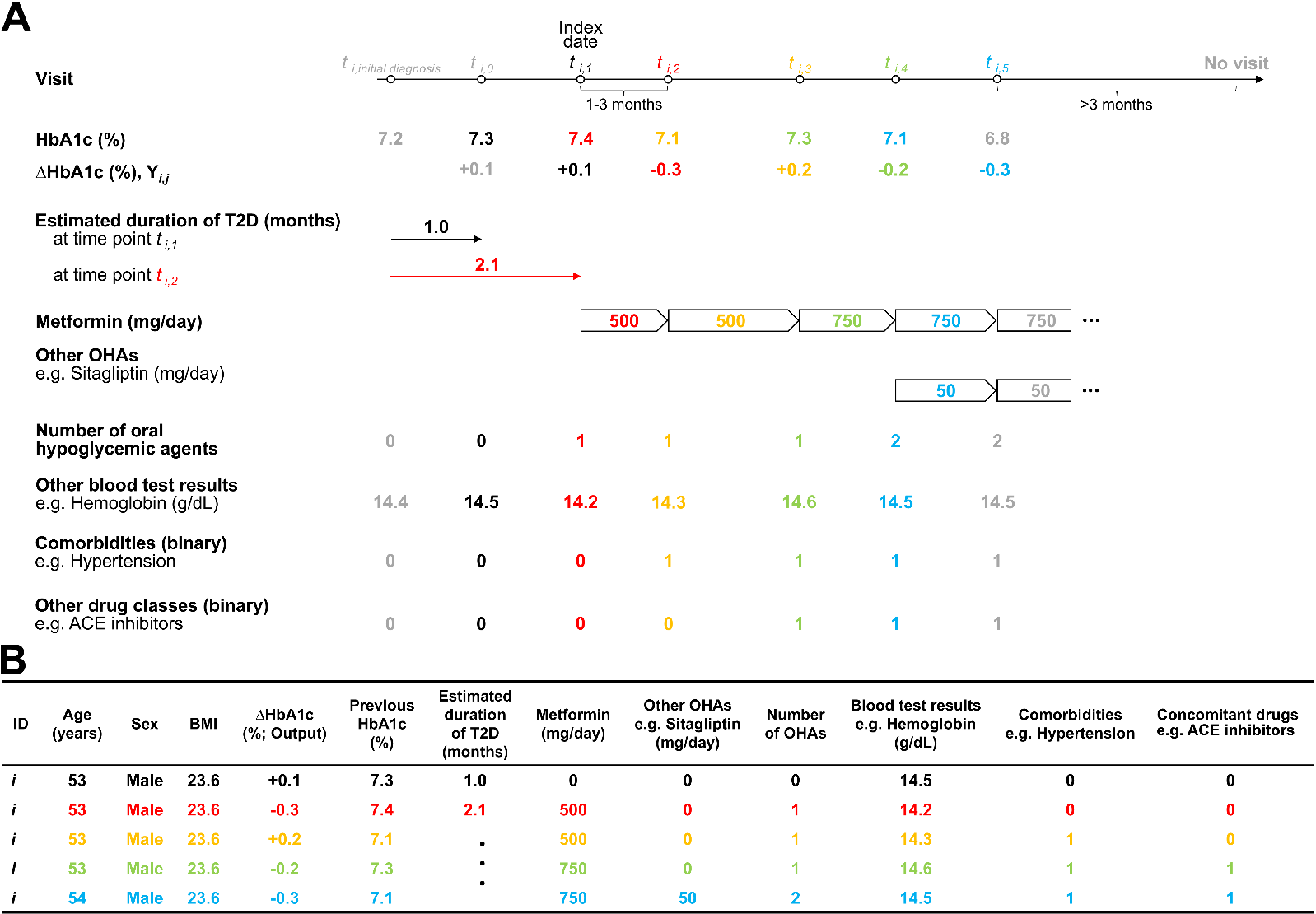


***Supplementary Figure S1. Visualization of irregular longitudinal data of Japanese patients with T2D.***

Panel A is clinical information of the *i*th patient with T2D followed up as long as he/she continued to attend at 1 to 3-month intervals. Panel B is long-format data processed from the clinical information of the *i*th patient. Data for each time point are color-coded to facilitate the reader’s understanding.


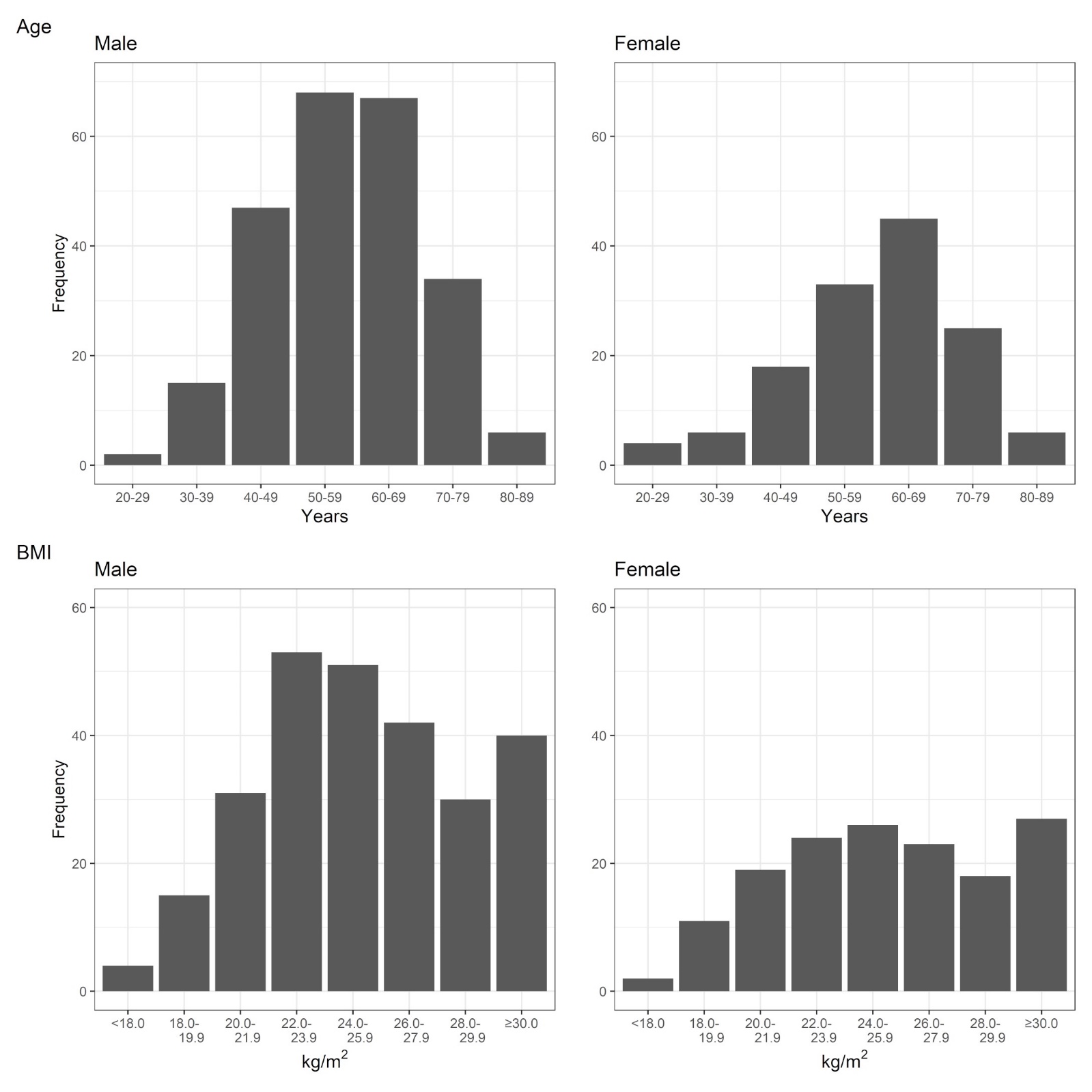


***Supplementary Figure S2. Age and BMI distribution of Japanese T2D patients at index date.***


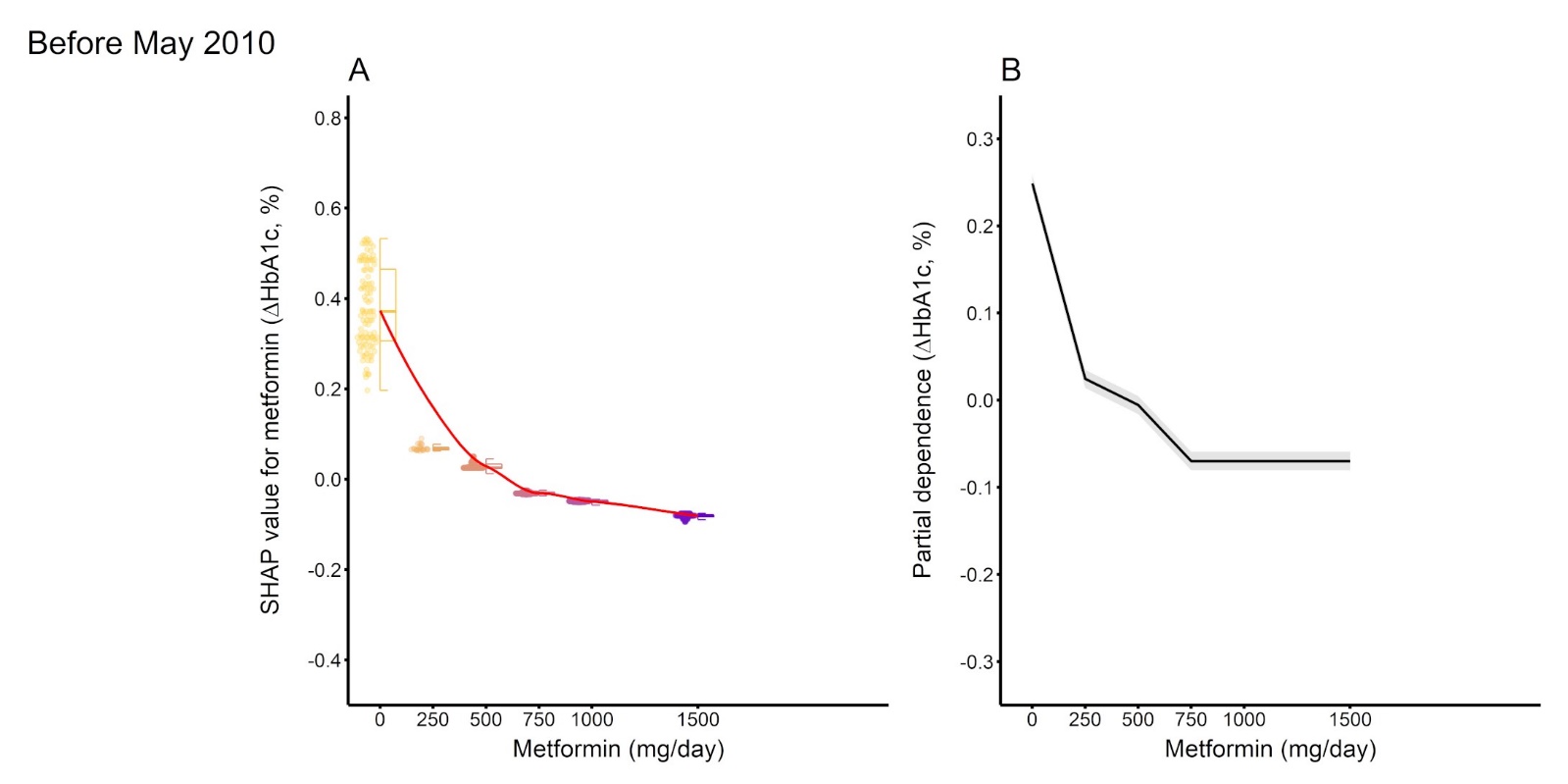


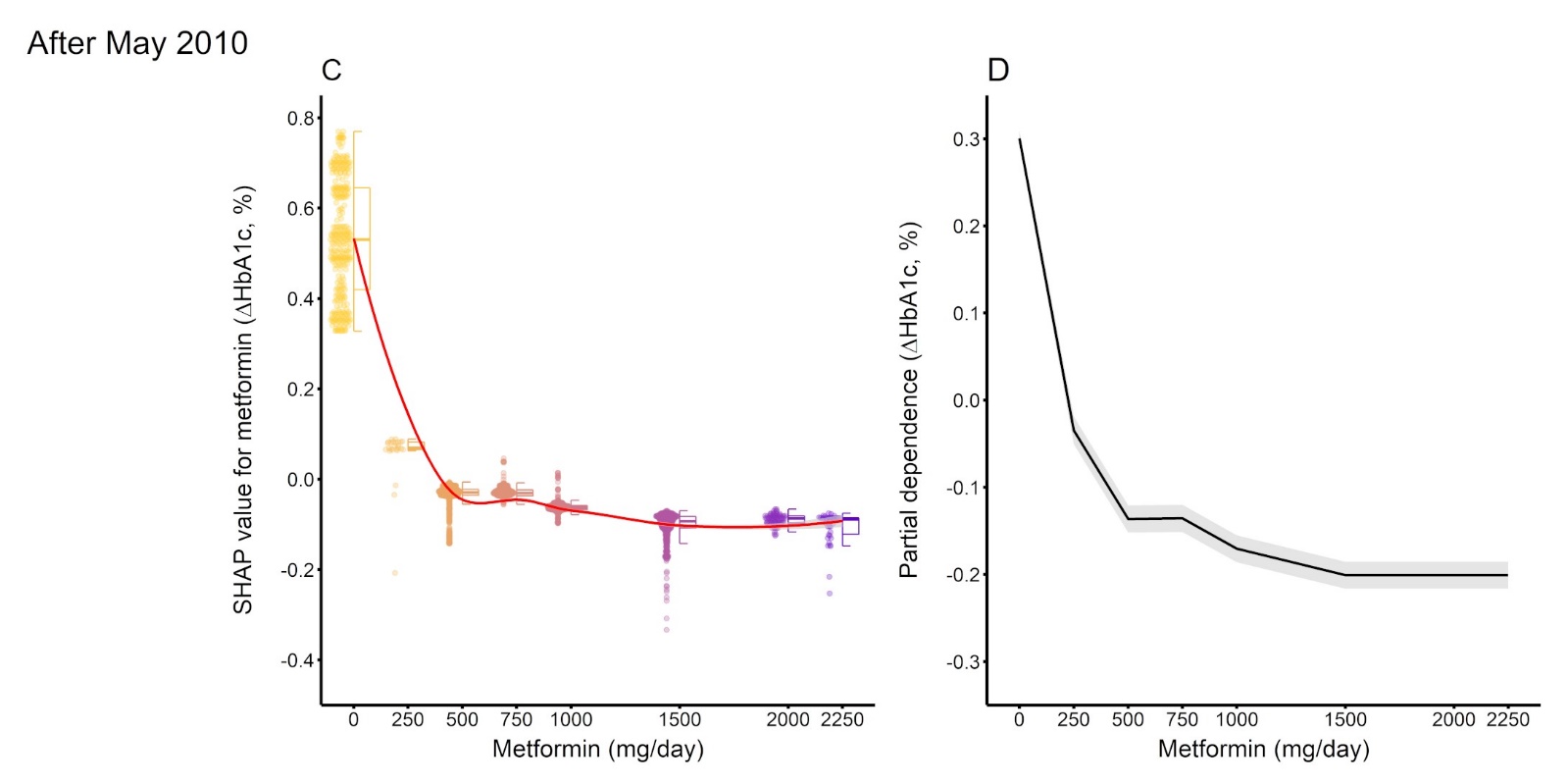


***Supplementary Figure S3. Dose-related response to metformin before and after May 2010.***

This shows the dose-response relationship between metformin and change in HbA1c level revealed by interpreting gpboost constructed from data split into before and after May 2010. Panels A and C show SHAP dependence plots. Panels B and D show partial dependence plots.


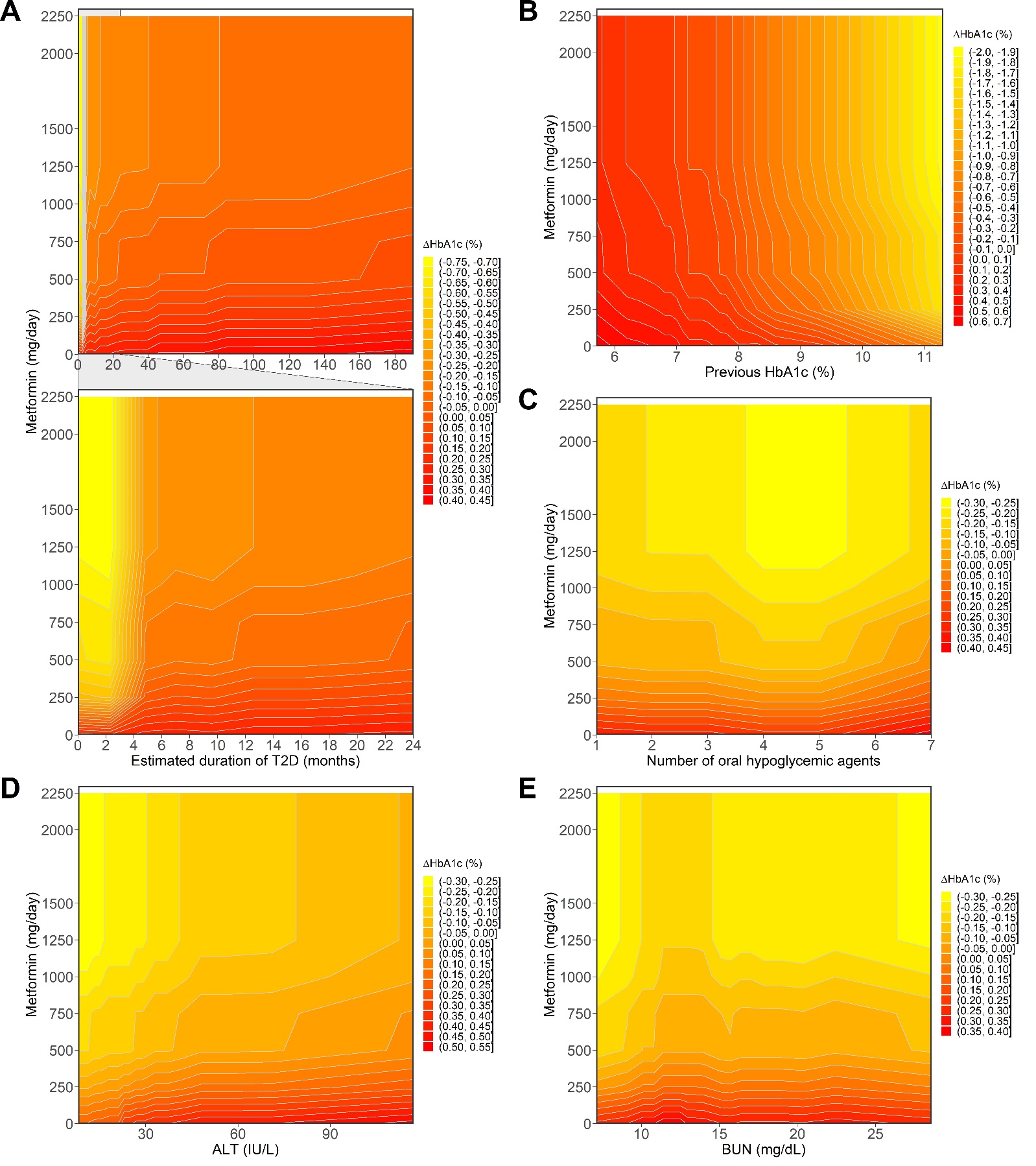


***Supplementary Figure S4. Interactions between daily dose of metformin and other continuous features.***

Panels are sorted in descending order of Friedman’s H statistic (Figure 5A).


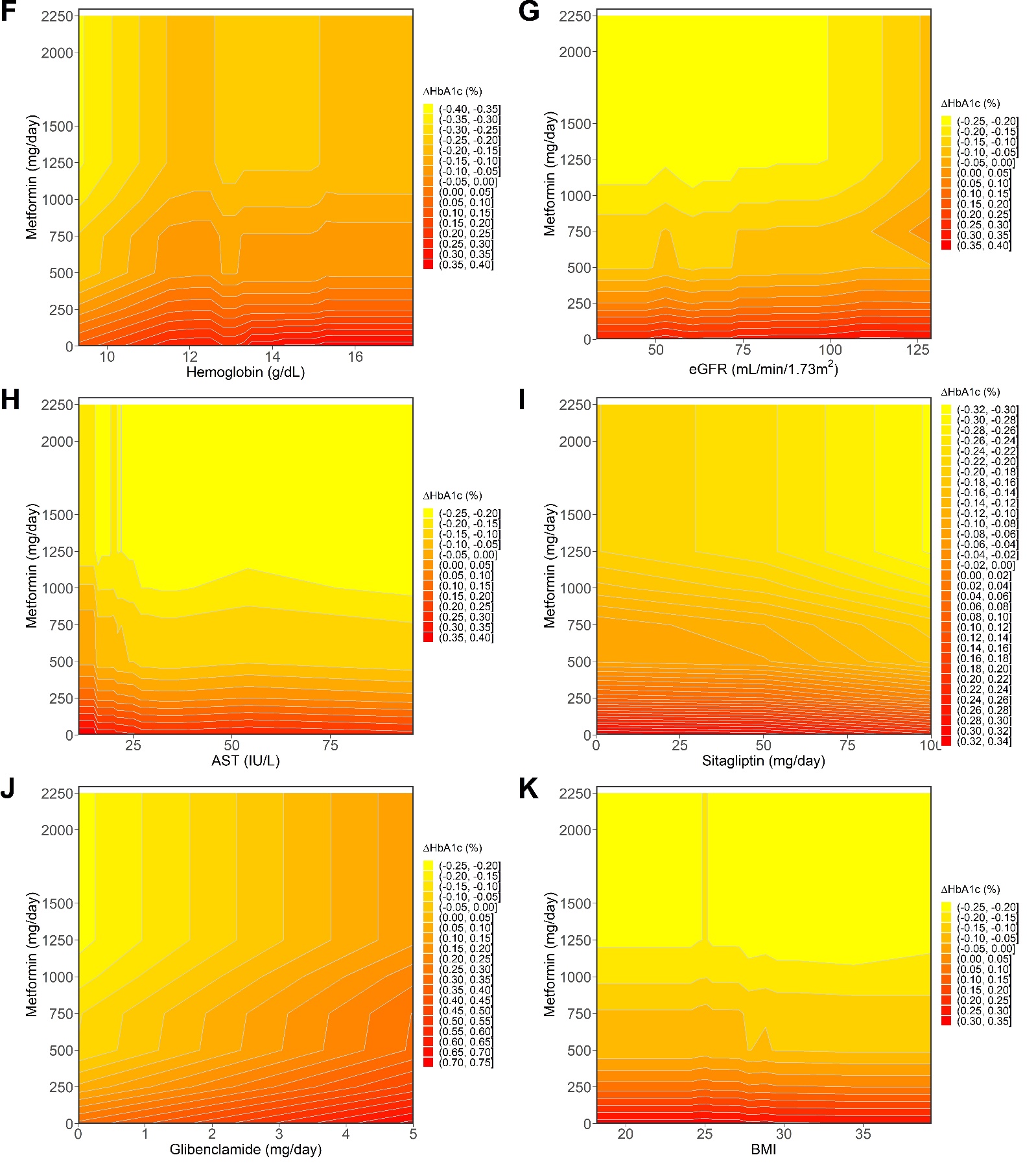


***Supplementary Figure S4. Continued.***


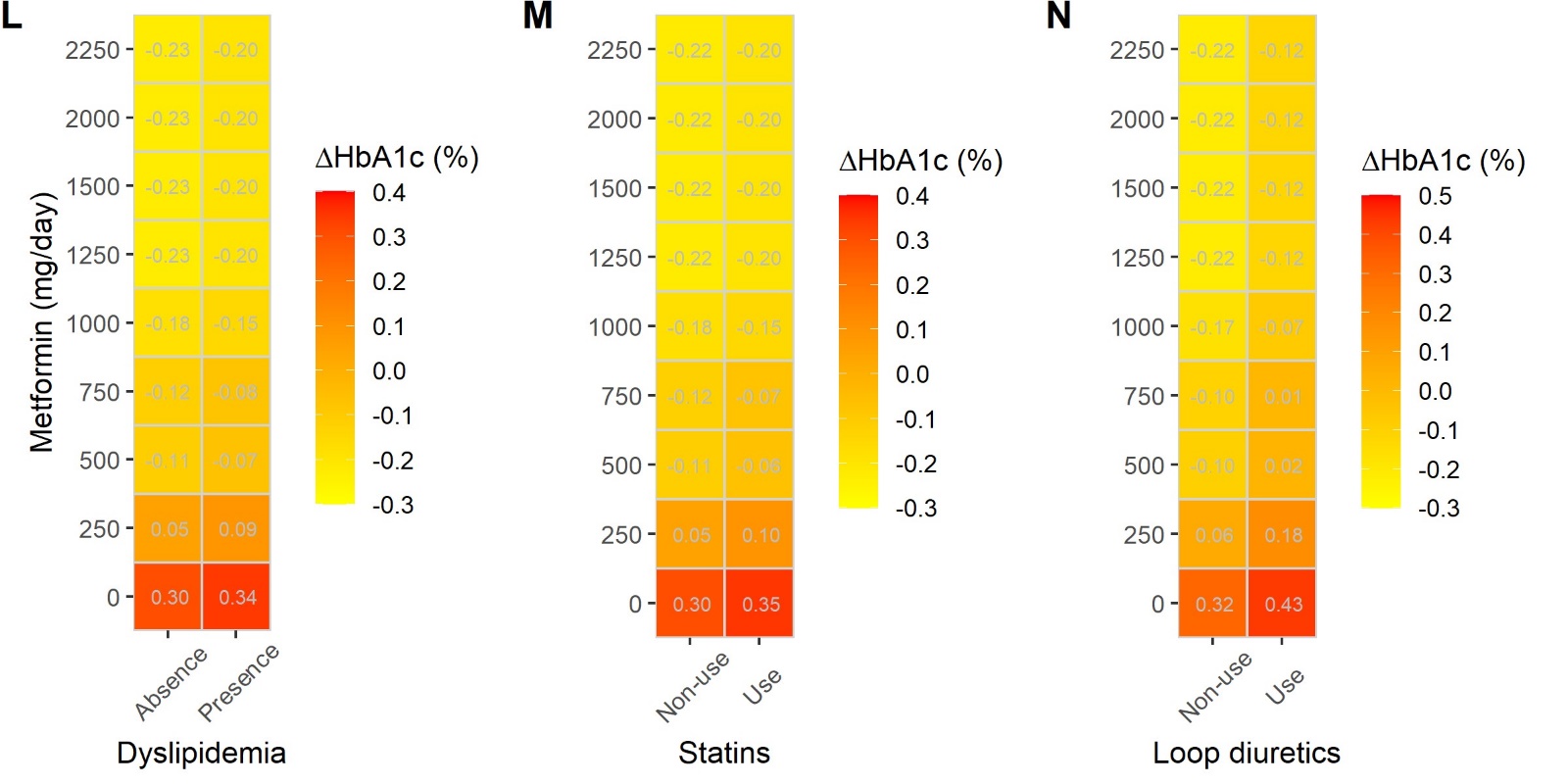


***Supplementary Figure S5. Interactions between daily dose of metformin and other binary features.***

Gray numbers in each cell are ΔHbA1c values corresponding to the figure legends, showing a gradient from red to yellow. For example, the HbA1c-lowering effect of metformin at 500 mg/day was 0.04 (%) greater in T2D patients without dyslipidemia than in those with dyslipidemia.
