# Supplementary material for: Non‐Linear Dose–Response Relationship for Metformin in Japanese Patients With Type 2 Diabetes: Analysis of Irregular Longitudinal Data by Interpretable Machine Learning Models
Source: Pharmacol Res Perspect. 2025 Feb 5;13(1):e70055. doi: 10.1002/prp2.70055 (PMC11797302; doi:10.1002/prp2.70055)
Supplement: Supplementary file 2 — Table S1. [file PRP2-13-e70055-s002.docx]

***Supplementary Tables***

**Non-linear dose-response relationship for metformin in Japanese patients with type 2 diabetes: Analysis of irregular longitudinal data by interpretable machine learning models**

**Hayato Akimoto^1,2^, Takuya Nagashima^1,2^, Kimino Minagawa^2^, Takashi Hayakawa^1,2^, Yasuo Takahashi^2^ and Satoshi Asai^1,2^**

* Correspondence: Hayato Akimoto: [akimoto.hayato@nihon-u.ac.jp](mailto:akimoto.hayato@nihon-u.ac.jp)

**Supplementary Table S1. International Classification of Disease 10 (ICD-10) codes.**

| **Disease** | **ICD-10** | |
| --- | --- | --- |
|  | **Code** | **Three-character category** |
| Type 2 diabetes | E11.X | Type 2 diabetes mellitus |
| Comorbidity |  |  |
| Hypertension | I10 | Essential (primary) hypertension |
| Dyslipidemia | E78.0, E78.1, E78.2, E78.5 | Disorders of lipoprotein metabolism and other lipidemias |
| Hyperthyroidism | E05.0, E05.9 | Thyrotoxicosis [hyperthyroidism] |
| Hypothyroidism | E03.9, E06.3 | Other hypothyroidism (E03), Thyroiditis (E06) |
| Chronic kidney disease | N18.1-5, N18.9 | Chronic kidney disease |
| Chronic liver disease | K72.1  K73.0, K73.2, K73.8, K73.9, K74.9, K76.9 | Hepatic failure, not elsewhere classified  Chronic hepatitis, not elsewhere classified |

**Supplementary Table S2. Anatomical Therapeutic Chemical (ATC) Classification of oral hypoglycemic agents.**

| **Oral hypoglycemic agents** | **Drug name (ATC 5^th^ level)** |
| --- | --- |
| Biguanides | metformin (A10BA02) |
| *As add-on treatment* |  |
| Alpha-glucosidase inhibitors | acarbose (A10BF01), miglitol (A10BF02), voglibose (A10BF03) |
| Glinides | repaglinide (A10BX02), nateglinide (A10BX03), mitiglinide (A10BX08) |
| Sulfonylureas | glibenclamide (A10BB01), gliclazide (A10BB09), glimepiride (A10BB12) |
| DPP-4 inhibitors | sitagliptin (A10BH01), vildagliptin (A10BH02), saxagliptin (A10BH03),  alogliptin (A10BH04), linagliptin (A10BH05), teneligliptin (A10BH08) |
| SGLT2 inhibitors | dapagliflozin (A10BK01), ipragliflozin (A10BK05), empagliflozin (A10BK03),  canagliflozin (A10BK02), luseogliflozin (A10BK07) |
| Thiazolidinediones | pioglitazone (A10BG03) |

Abbreviations: DPP-4, dipeptidyl peptidase-4; SGLT2, sodium-glucose cotransporter 2

**Supplementary Table S3. Anatomical Therapeutic Chemical (ATC) Classification of other drugs**

| **Drug class** | **Drug name (ATC 5^th^ level)** |
| --- | --- |
| ACE inhibitors | captopril (C09AA01), enalapril (C09AA02), lisinopril (C09AA03), perindopril (C09AA04), quinapril (C09AA06), benazepril (C09AA07), cilazapril (C09AA08), trandolapril (C09AA10), delapril (C09AA12), temocapril (C09AA14), imidapril (C09AA16), alacepril (none) |
| ARBs | losartan (C09CA01), valsartan (C09CA03), irbesartan (C09CA04), candesartan (C09CA06), telmisartan (C09CA07), olmesartan (C09CA08), azilsartan (C09CA09) |
| Calcium channel blockers | amlodipine (C08CA01), felodipine (C08CA02), nicardipine (C08CA04), nifedipine (C08CA05), nisoldipine (C08CA07), nitrendipine (C08CA08), aranidipine (none), efonidipine (none), nilvadipine (C08CA10), manidipine (C08CA11), barnidipine (C08CA12), benidipine (C08CA15), azelnidipine (none), Cilnidipine (none) |
| α blockers | prazosin (C02CA01), doxazosin (C02CA04), urapidil (C02CA06), terazosin (G04CA03), bunazosin (none) |
| Selective β_1_ blockers | metoprolol (C07AB02), atenolol (C07AB03), acebutolol (C07AB04), betaxolol (C07AB05), bisoprolol (C07AB07), celiprolol (C07AB08), |
| Loop diuretics | furosemide (C03CA01), bumetanide (C03CA02), torasemide (C03CA04), azosemide (none) |
| Thiazide diuretics | trichlormethiazide (C03AA06), hydrochlorothiazide (C03AA03), benzylhydrochlorothiazide (none) |
| Statins | simvastatin (C10AA01), pravastatin (C10AA03), fluvastatin (C10AA04), atorvastatin (C10AA05), rosuvastatin (C10AA07), pitavastatin (C10AA08) |
| Fibrates | clofibrate (C10AB01), bezafibrate (C10AB02), fenofibrate (C10AB05), pemafibrate (C10AB12), clinofibrate (none) |
| Steroids for systemic use | betamethasone (H02AB01), dexamethasone (H02AB02), methylprednisolone (H02AB04), prednisolone (H02AB06), triamcinolone (H02AB08), hydrocortisone (H02AB09), cortisone (H02AB10) |
| SDAs | risperidone (N05AX08), paliperidone (N05AX13) |
| MARTAs (excluded) | clozapine (N05AH02), olanzapine (N05AH03), quetiapine (N05AH04) |

Drug classes except for MARTAs were treated as binary features in analyses.

Abbreviations: ACE, angiotensin converting enzyme; ARB, angiotensin receptor blocker; MARTA, multi-acting receptor-targeted antipsychotic; SDA, serotonin dopamine antagonist

**Supplementary Table S4. Optimized hyperparameters in combining tree-boosting with Gaussian process and mixed-effects models and predictive performance.**

|  | **Default** | **Optimized** |
| --- | --- | --- |
| Hyperparameters |  |  |
| learning rate | 0.1 | 0.1 |
| max_depth ^†^ | -1 | 2 |
| num_leaves | 31 | 5 |
| min_data_in_leaf | 20 | 10 |
| lambda_l2 | 0 | 177.83 |
| Nrounds | 100 | 169 |
| RMSE (95%CI) ^‡^ |  |  |
| Training data | 0.224 (0.219-0.228) | 0.464 (0.448-0.481) |
| Testing data | 0.806 (0.726-0.895) | 0.602 (0.523-0.684) |

Grid search space is as follows:

learning rate: {0.01, 0.1, 1}

max_depth: {2, 4, 6, 8, 10}

num_leaves: {ranged from 5 to 50 by 5}

min_data_in_leaf: {ranged from 10 to 100 by 10}

lambda_l2: {10^(ranged from -3 to 3 by 0.25)}

† Max_depth ≤0 means no limit.

‡ 95%CI was estimated by percentile bootstrap method.

Abbreviations: 95%CI, 95% confidence interval; RMSE, root mean square error

**Supplementary Table S5. Regression coefficients of all 50 features in two linear algorithms.**

| Features | GLMM | | | | |  | Feature-selected GLMM | | | | |
| --- | --- | --- | --- | --- | --- | --- | --- | --- | --- | --- | --- |
|  | Coefficient | Standard error | Df | t value | p value |  | Coefficient | Standard error | df | t value | p value |
| Age [/1 year] | 0.009 | 0.023 | 309 | 0.421 | 0.674 |  | - | - | - | - | - |
| Male | 0.155 | 0.050 | 256 | 3.069 | 0.002 |  | 0.137 | 0.048 | 253 | 2.839 | 0.005 |
| BMI | 0.001 | 0.005 | 245 | 0.162 | 0.872 |  | - | - | - | - | - |
| Estimated duration of T2D [/1 month] | 0.074 | 0.009 | 108 | 8.032 | <0.001 |  | 0.075 | 0.009 | 106 | 8.607 | <0.001 |
| Previous HbA1c | -0.394 | 0.009 | 5203 | -46.218 | <0.001 |  | -0.388 | 0.008 | 5146 | -46.468 | <0.001 |
| Hemoglobin | 0.052 | 0.010 | 2611 | 5.355 | <0.001 |  | 0.050 | 0.009 | 2291 | 5.361 | <0.001 |
| AST | -0.003 | 0.001 | 6113 | -3.342 | <0.001 |  | -0.002 | 0.001 | 6062 | -3.095 | 0.002 |
| ALT | 0.004 | 0.001 | 5181 | 4.878 | <0.001 |  | 0.003 | 0.001 | 4900 | 4.690 | <0.001 |
| BUN | 0.003 | 0.002 | 5726 | 1.330 | 0.184 |  | - | - | - | - | - |
| eGFR | 0.003 | 0.001 | 2005 | 3.418 | <0.001 |  | 0.002 | 0.001 | 1440 | 2.928 | 0.003 |
| Number of oral hypoglycemic drugs | -0.073 | 0.030 | 2644 | -2.463 | 0.014 |  | -0.066 | 0.016 | 1533 | -4.245 | <0.001 |
| Metformin [/500 mg/day] | -0.234 | 0.014 | 3994 | -16.176 | <0.001 |  | -0.231 | 0.014 | 3894 | -16.314 | <0.001 |
| Acarbose | 0.000 | 0.001 | 589 | 0.436 | 0.663 |  | - | - | - | - | - |
| Voglibose | -0.044 | 0.107 | 1723 | -0.411 | 0.681 |  | - | - | - | - | - |
| Miglitol | 0.000 | 0.000 | 1596 | 0.294 | 0.769 |  | - | - | - | - | - |
| Alogliptin | 0.003 | 0.003 | 2210 | 0.814 | 0.416 |  | - | - | - | - | - |
| Saxagliptin | 0.048 | 0.026 | 1155 | 1.816 | 0.070 |  | - | - | - | - | - |
| Sitagliptin | -0.002 | 0.001 | 1752 | -2.020 | 0.044 |  | -0.002 | 0.001 | 1668 | -2.673 | 0.008 |
| Teneligliptin | 0.003 | 0.003 | 1954 | 1.320 | 0.187 |  | - | - | - | - | - |
| Vildagliptin | -0.001 | 0.000 | 2499 | -2.288 | 0.022 |  | -0.001 | 0.000 | 2276 | -2.987 | 0.003 |
| Linagliptin | -0.007 | 0.015 | 2729 | -0.486 | 0.627 |  | - | - | - | - | - |
| Nateglinide | -0.001 | 0.001 | 2100 | -1.337 | 0.181 |  | - | - | - | - | - |
| Mitiglinide | 0.013 | 0.005 | 2778 | 2.614 | 0.009 |  | 0.012 | 0.004 | 2356 | 2.769 | 0.006 |
| Repaglinide | 0.200 | 0.146 | 1360 | 1.373 | 0.170 |  | - | - | - | - | - |
| Ipragliflozin | -0.003 | 0.002 | 2422 | -1.697 | 0.090 |  | - | - | - | - | - |
| Empagliflozin | -0.008 | 0.009 | 2191 | -0.959 | 0.338 |  | - | - | - | - | - |
| Canagliflozin | -0.002 | 0.001 | 3337 | -2.639 | 0.008 |  | -0.001 | 0.001 | 2665 | -2.278 | 0.023 |
| Dapagliflozin | -0.007 | 0.011 | 2233 | -0.617 | 0.537 |  | - | - | - | - | - |
| Luseogliflozin | -0.013 | 0.068 | 1538 | -0.194 | 0.846 |  | - | - | - | - | - |
| Glimepiride | 0.005 | 0.019 | 3616 | 0.266 | 0.791 |  | - | - | - | - | - |
| Gliclazide | 0.002 | 0.001 | 1454 | 1.272 | 0.204 |  | 0.002 | 0.001 | 1051 | 1.365 | 0.173 |
| Glibenclamide | 0.092 | 0.017 | 1674 | 5.558 | <0.001 |  | 0.089 | 0.015 | 1461 | 5.873 | <0.001 |
| Pioglitazone | -0.003 | 0.003 | 2068 | -0.925 | 0.355 |  | - | - | - | - | - |
| Hypertension | -0.127 | 0.045 | 1634 | -2.822 | 0.005 |  | -0.124 | 0.043 | 1531 | -2.909 | 0.004 |
| Dyslipidemia | -0.074 | 0.075 | 1342 | -0.986 | 0.324 |  | - | - | - | - | - |
| Hyperthyroidism | -0.096 | 0.138 | 449 | -0.691 | 0.490 |  | - | - | - | - | - |
| Hypothyroidism | -0.176 | 0.112 | 401 | -1.562 | 0.119 |  | - | - | - | - | - |
| Chronic liver disease | -0.113 | 0.123 | 399 | -0.916 | 0.360 |  | - | - | - | - | - |
| Chronic kidney disease | 0.069 | 0.102 | 563 | 0.678 | 0.498 |  | - | - | - | - | - |
| ARBs | 0.127 | 0.040 | 2520 | 3.197 | 0.001 |  | 0.134 | 0.039 | 2359 | 3.467 | <0.001 |
| ACE inhibitors | -0.078 | 0.084 | 1077 | -0.931 | 0.352 |  | - | - | - | - | - |
| Calcium channel blockers | -0.117 | 0.041 | 2090 | -2.878 | 0.004 |  | -0.114 | 0.039 | 2098 | -2.906 | 0.004 |
| Selective β_1_ blockers | 0.021 | 0.087 | 581 | 0.235 | 0.814 |  | - | - | - | - | - |
| α blockers | -0.022 | 0.087 | 1513 | -0.249 | 0.803 |  | - | - | - | - | - |
| Loop diuretics | 0.280 | 0.064 | 1981 | 4.383 | <0.001 |  | 0.267 | 0.059 | 1687 | 4.545 | <0.001 |
| Thiazide diuretics | 0.139 | 0.091 | 1200 | 1.527 | 0.127 |  | - | - | - | - | - |
| Statins | 0.113 | 0.076 | 1348 | 1.493 | 0.136 |  | - | - | - | - | - |
| Fibrates | 0.083 | 0.067 | 2465 | 1.234 | 0.217 |  | - | - | - | - | - |
| SDAs | -0.135 | 0.126 | 484 | -1.071 | 0.285 |  | - | - | - | - | - |
| Steroids (systemic use) | -0.051 | 0.049 | 1165 | -1.026 | 0.305 |  | - | - | - | - | - |

Abbreviations: ACE, angiotensin converting enzyme; ALT, alanine aminotransferase: ARB, angiotensin receptor blocker; AST, aspartate aminotransferase; BUN, blood urea nitrogen; df, degrees of freedom; eGFR, estimated glomerular filtration rate; GLMM, generalized linear mixed effects model; HbA1c, hemoglobin A1c; SDA, serotonin dopamine antagonist, T2D, type 2 diabetes
